# Tetracyclic Thioxanthene Derivatives: Studies on Fluorescence and Antitumor Activity

**DOI:** 10.3390/molecules26113315

**Published:** 2021-05-31

**Authors:** Fernando Durães, Patrícia M. A. Silva, Pedro Novais, Isabel Amorim, Luís Gales, Cátia I. C. Esteves, Samuel Guieu, Hassan Bousbaa, Madalena Pinto, Emília Sousa

**Affiliations:** 1 Laboratory of Organic and Pharmaceutical Chemistry, Department of Chemical Sciences, Faculty of Pharmacy, University of Porto, Rua de Jorge Viterbo Ferreira, 228, 4050-313 Porto, Portugal; fduraes5@gmail.com (F.D.); madalena@ff.up.pt (M.P.); 2 CIIMAR-Interdisciplinary Centre of Marine and Environmental Research, University of Porto, Novo Edifício do Terminal de Cruzeiros do Porto de Leixões, Avenida General Norton de Matos s/n, 4450-208 Matosinhos, Portugal; 3 CESPU, Institute of Research and Advanced Training in Health Sciences and Technologies (IINFACTS), Rua Central de Gandra, 1317, 4585-116 Gandra, Portugal; patricia.silva@cespu.pt (P.M.A.S.); pedro.ha.novais@gmail.com (P.N.); 4 Department of Biochemistry, Faculty of Sciences, University of Porto, Rua do Campo Alegre s/n, 4169-007 Porto, Portugal; 5 GreenUPorto (Sustainable Agrifood Production) Research Center, Faculty of Sciences, University of Porto, Rua do Campo Alegre s/n, 4169-007 Porto, Portugal; mpamorim@fc.up.pt; 6 Department of Molecular Biology, ICBAS-Instituto de Ciências Biomédicas Abel Salazar, University of Porto, Rua de Jorge Viterbo Ferreira, 228, 4050-313 Porto, Portugal; lgales@ibmc.up.pt; 7 Bioengineering & Synthetic Microbiology, I3S–Instituto de Investigação e Inovação em Saúde, University of Porto, Rua Alfredo Allen 208, 4200-135 Porto, Portugal; 8 LAQV-REQUIMTE, Department of Chemistry, University of Aveiro, 3810-193 Aveiro, Portugal; catiaiesteves@ua.pt (C.I.C.E.); sguieu@ua.pt (S.G.); 9 Department of Chemistry, CICECO-Aveiro Institute of Materials, University of Aveiro, 3810-193 Aveiro, Portugal

**Keywords:** thioxanthenes, theranostic, antitumor activity, photophysics

## Abstract

Thioxanthones are bioisosteres of the naturally occurring xanthones. They have been described for multiple activities, including antitumor. As such, the synthesis of a library of thioxanthones was pursued, but unexpectedly, four tetracyclic thioxanthenes with a quinazoline–chromene scaffold were obtained. These compounds were studied for their human tumor cell growth inhibition activity, in the cell lines A375-C5, MCF-7 and NCI-H460. Photophysical studies were also performed. Two of the compounds displayed GI_50_ values below 10 µM for the three tested cell lines, and structure–activity relationship studies were established. Three compounds presented similar wavelengths of absorption and emission, characteristic of dyes with a push-pull character. The structures of two compounds were elucidated by X-ray crystallography. Two tetracyclic thioxanthenes emerged as hit compounds. One of the two compounds accumulated intracellularly as a bright fluorescent dye in the green channel, as analyzed by both fluorescence microscopy and flow cytometry, making it a promising theranostic cancer drug candidate.

## 1. Introduction

**Figure 1:**
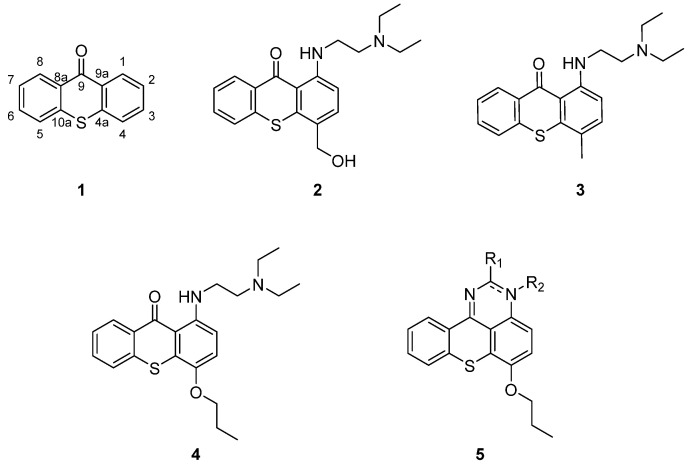
Structure of the thioxanthone scaffold (**1**), the anticancer thioxanthones hycanthone (**2**) and lucanthone (**3**), the dual tumor cell line growth/P-glycoprotein inhibitor (**4**) and the general structure of the compounds studied in this work (**5**).

Thioxanthones have sparked interest in terms of medicinal chemistry, due to the fact that they are bioisosteres of xanthones, a privileged structure with occurrence in natural products [1,2]. They are *S*-heterocycles, with three rings and a dibenzo-γ-pyrone scaffold (**1**, Figure 1). This class of heterocycles has gained increasing importance in medicinal chemistry, due to its versatility and presence in bioactive compounds in a wide range of biological targets. Therefore, the substitution of a natural scaffold with a sulfur opens the gate for the conception of small molecules with a potential large array of activity [3]. The properties of the classical bivalent isosteres CH_2_, O and S, such as electronegativity, log P, bond length and distance, van der Waals radius and volume, reveal some similarities and differences that have proven their utility in drug design [4]. Allied to the chemical versatility of the sulfur atom, these physicochemical differences contribute to making this scaffold attractive for the development of new molecules. 

Recent studies performed by our group have proven the usefulness of thioxanthones in a multitude of diverse activities. A library of thioxanthones was tested for their antimicrobial and synergistic activity, proving the potential of these compounds to inhibit the growth of pathogenic bacteria and to revert antimicrobial resistance [5]. They have also proven successful in reducing lipids, with no associated toxicity in the tested model, uncovering potential anti-obesity activity [6]. Moreover, thioxanthones have been investigated in the last decades for their antitumor activity [7,8,9,10] and have also been studied for their photoinitiator features [11,12,13,14]. Hycanthone (**2**, Figure 1) and its prodrug, lucanthone (**3**, Figure 1), were the first thioxanthones introduced in therapy as antischistosomal agents [1,15,16]. However, these drugs led to mutagenicity, likely associated with their C-4 methylene moiety, which resulted in their removal from therapeutics [17]. This effect led to the discovery of their ability to sensitize cancer cells, suggesting new and promising applications in chemotherapy. Previous results by our group found that aminated thioxanthone **4** (Figure 1), structurally similar to **2** and **3**, is a potent cell growth inhibitor in the myeloid leukemia K562 cell line, presenting GI_50_ values of 1.9 µM for both the doxorubicin-sensitive and resistant cell line. Regarding solid tumors, compound **4** also emerged as a hit compound for the three tested cell lines: MCF-7 (breast adenocarcinoma), NCI-H460 (non-small cell lung cancer) and A375-C5 (melanoma), displaying the best results out of the library of thioxanthones tested [18]. Deeper insights into the mechanism revealed compound **4** as capable of modulating autophagy and inducing apoptosis, thus becoming a lead compound for tumor treatment, with proven efficiency in mice xenografts [10,19]. Furthermore, **4** also displayed inhibition of P-glycoprotein, a known efflux pump, working as a dual antitumor/P-glycoprotein inhibitor agent with improved efficacy in sensitizing a resistant P-gp overexpressing cell line to doxorubicin [7,20]. 

Theranostic, the combination of therapy and diagnostic, has been gathering importance, particularly in cancer, through the application of molecular imaging towards the identification of cancer targets and the optimization of the therapy. Specifically, these agents concomitantly foster the transport of both therapeutic and diagnostic imaging agents, therefore reducing the biodistribution and therapeutic efficacy differences between them [21]. The focus of this work is the synthesis and biological evaluation of novel aminated thioxanthones based on the lead compound **4** as tumor cell line inhibitors and preliminary studies to understand their potential for theranostics. For that purpose, the amines chosen were guanidine and urea derivatives to allow a tandem reaction with further C-9 ketone functionalization which yielded tetracyclic derivatives. This tetracyclic scaffold is based on the D-π-A structure (“push-pull” system), architected by an electron donor group (C-4 propoxy chain), an electron acceptor group (C-1 guanidine/urea) and bridged by a conjugated π system (**5**, Figure 1). Most of the near-infrared fluorescence (NIR) probes are based on this structure, combining a rigid planarity capability and beneficially shifting the emission wavelength into the NIR region. Indeed, during the development of this work, new tetracyclic thioxanthenes, with a quinazoline–chromene scaffold, were synthesized and described as potential antitumor agent kinase inhibitors [8,22], while three other new thioxanthenes were revealed to be promising as theranostic agents [23,24,25,26], highlighting the potential of the rationale herein followed.

## 2. Results and Discussion

### 2.1. Chemistry

#### Synthesis of Aminated Tetracyclic Thioxanthenes

**Scheme 1:**
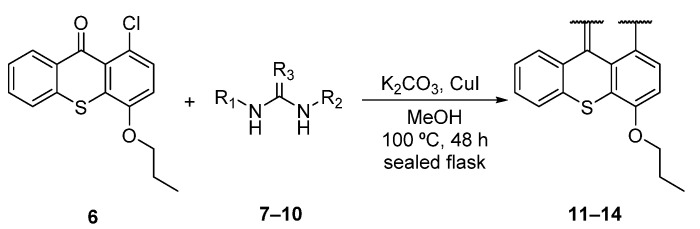
Synthetic pathway towards obtaining tetracyclic thioxanthenes.

Thioxanthenes **11**–**14** were synthesized from 1-chloro-4-propoxy-9*H*-thioxanthen-9-one (**6**) by nucleophilic aromatic substitution (Ullmann type C–N coupling) of the chlorine in position C-1 by a secondary amine (**7**–**10**). A dehydrative cyclization happens in a concerted manner, leading to the formation of a tetracyclic structure [27]. Scheme 1 shows the general reaction for the synthesis of aminated thioxanthenes, and Table 1 shows the amines and thioxanthenes obtained.

**Scheme 2:**
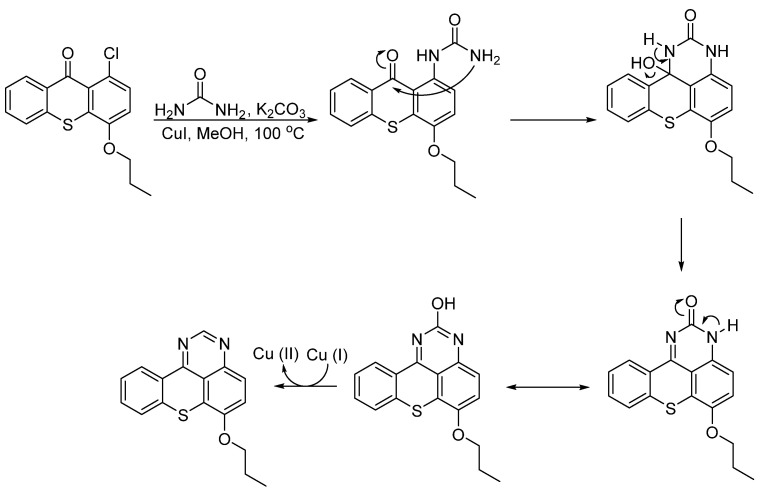
Proposed reaction mechanism for the cyclization of compound **12**.

Noteworthy is also the fact that, in the case of thioxanthene **12**, a reduction occurs, probably mediated by copper iodide, yielding the pyrimidine-like fourth ring, instead of the expected aromatic ring substituted with a carbonyl [28]. The proposed reaction mechanism for the cyclization and reduction, leading to compound **12**, is presented in Scheme 2.

### 2.2. Biological Activity

#### Antitumor Activity

The synthesized compounds were tested for their capability of inhibiting the growth of human tumor cell lines. The tetracyclic thioxanthenes herein described show potential as antitumor agents for the tested cell lines, particularly **11** and **14**, which have emerged as hit compounds, presenting concentrations required to reduce growth rates to 50% (GI_50_) in A375-C5 (IL-1 insensitive malignant melanoma), MCF-7 (breast adenocarcinoma) and NCI-H460 (non-small cell lung cancer) of 5–7 µM and 8–11 µM, respectively. Compound **12** presented concentrations between 31 and 39 µM for the tested cell lines and **13** displayed GI_50_ between 16 and 23 µM. The results are presented in Table 2.

We performed cell toxicity study of the compounds using non-tumor cell lines. We found that the GI_50_ of the four compounds in the non-tumor Human Pulmonary Alveolar Epithelial Cells (HPAEpiC) was higher than that in cancer cell lines under study (Table 3). The compounds exhibited a high degree of selectivity, as determined by selectivity index calculation, suggesting that they are not toxic to healthy cells, at least at concentrations that are toxic to cancer cells. Interestingly, compounds **11** and **13** exhibited a better degree of selectivity than doxorubicin.

Overall, compound **11** showed the best GI_50_ values for all the tested cell lines, meaning this could be a starting point for the development of new antitumor compounds. This compound is the only one bearing a free amino group, which makes it easier for it to pass negatively charged cell membranes. This is evidenced by the much different GI_50_ between this compound and the structurally related **12**, which is substituted with a hydrogen and presents much lower activity. Substitution of the free amino group with a 2-chlorobenzyl moiety (compound **13**) also leads to lower activity in the tested cell lines. The imine derivative **14** presented activity comparable to **11**, but had slightly higher GI_50_. This can suggest that substitution of the free amino group does not necessarily lead to lack of activity, but synthesis of different derivatives will lead to clearer conclusions over structure–activity relationships.

### 2.3. Fluorescence

**Figure 2:**
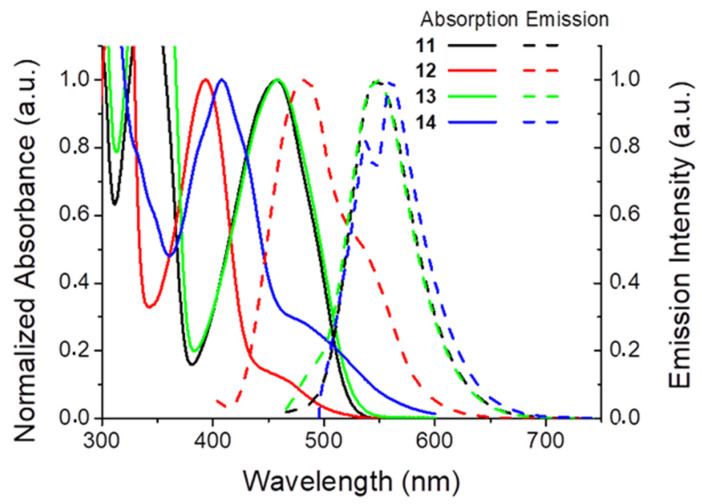
Normalized absorption and emission spectra DMSO (concentrations: **11**, 5 × 10^−5^ M; **12**, 1 × 10^−5^ M; **13**, 5 × 10^−5^ M; **14**, 1 × 10^−4^ M).

The absorption and emission spectra of all compounds were recorded in diluted DMSO solutions (Figure 2), and their main characteristics are summarized in Table 4.

Compounds **11**, **13** and **14** present similar wavelengths of absorption and emission, with a large Stokes’ shift characteristic of dyes with a push-pull character. When the nitrogen is absent from the position 1′, as in compound **12**, both absorption and emission are blue shifted. This nitrogen therefore seems to have an important contribution to the push-pull character of the dyes. On the other hand, the best quantum yield is obtained for compound **12**, lacking this nitrogen atom, which therefore seems detrimental to the emission intensity. Moreover, compound **14**, in which the aromaticity is disrupted because the nitrogen bears an extra group, is barely emissive in solution. Both the aromaticity of the heterocycle and the electron donating nature of the substituent seem important to obtain a high quantum yield and a large Stokes’ shift.

### 2.4. Fluorescence Microscopy and Flow Cytometry Analysis

**Figure 3:**
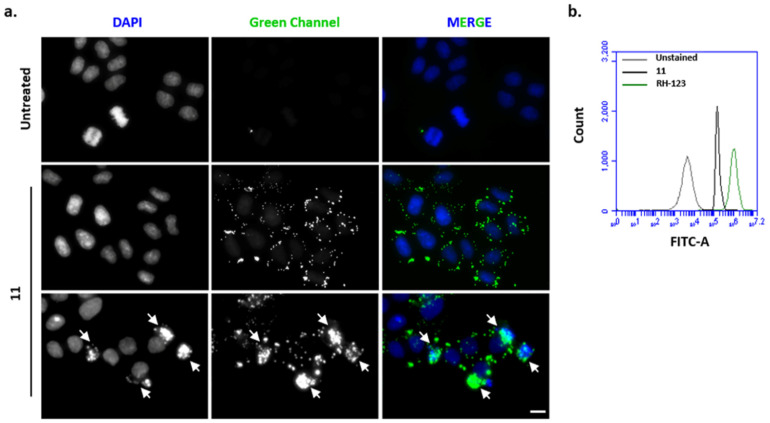
Cancer cells treated with compound **11** emitted fluorescence in the green channel. (**a**) Representative images of untreated (top) and compound **11**-treated cells (middle and bottom), showing intracellular green fluorescence as emitted by the compound. Middle: representative microscopic field showing (still) living cells treated with the compound: intact nuclei can be seen after DAPI staining of DNA (blue); in the green channel, fluorescence staining of the compound is seen in the cytoplasm, excluding the nuclei. Bottom: representative microscopic field showing treated cells undergoing cell death (white arrows): micronuclei can be seen after DAPI staining (blue), and a bright fluorescence is seen due to chromatin overcondensation and shrinking of dead cells. Bar, 5 µm. (**b**) Representative flow cytometry histogram of cell count versus FITC-A intensity in untreated (unstained) and compound **11**-treated cells. Rhodamine-123 green fluorescent dye was used as positive control.

A fluorescence microscopy analysis was undertaken to check the brightness of the four compounds in culture cells. The analysis revealed that compound **11** emitted a bright intracellular fluorescence in the green channel, when cells were excited with 470 nm light, in the cancer cells tested (Figure 3a). 

The fluorescence staining accumulated within intracellular round-shaped structures that closely resemble endosome/lysosome structures, which led us to suggest that the component may accumulate in those structures. However, this was not confirmed by the colocalization study using the lysosome marker LAMP-1 antibody (data not shown). The fluorescence staining remained associated with the cells until cell death. The fluorescence property of the compound **11** was confirmed when treated cells were analyzed by flow cytometry (Figure 3b). A peak of fluorescent cells was obtained in the green channel, near the peak of Rhodamine 123, used as positive control. Moreover, the fluorescence was stable after keeping the cell preparations in the refrigerator for more than two weeks. Additional studies are needed to elucidate the exact subcellular localization of compound **11** and its significance in terms of therapeutic application.

### 2.5. X-ray Crystallography

**Figure 4:**
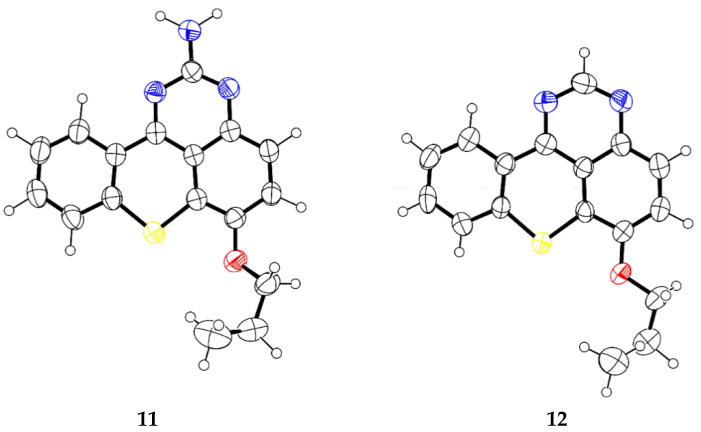
ORTEP view of compounds **11** and **12**.

The structures of compounds **11** and **12** were determined by single crystal X-ray diffraction (Figure 4). We have previously shown that while the most stable structure of tetrahydrothio-γ-pyrone is a chair conformation, thioxanthone molecules, like xanthones in general [30], are essentially planar [31]. The four-ring systems of **11** and **12** are also almost planar as expected. The central pyranoid rings have partial aromatic character with C-S bond lengths (from 1.741(4) to 1.752(4) Å) shorter than the corresponding bonds in the tetrahydrothio-γ-pyrone (1.806(4) and 1.814(4) Å) [31].

## 3. Materials and Methods

### 3.1. Chemistry

All reagents and solvents were purchased from Sigma Aldrich (Sigma-Aldrich Co. Ltd., Gillinghan, UK) and no further purification process was implemented. Solvents were evaporated using a rotary evaporator under reduced pressure, Buchi Waterchath B-480. All reactions were monitored by thin-layer chromatography (TLC) carried out on precoated plates with 0.2 mm of thickness using Merck silica gel 60 (GF_254_) with appropriate mobile phases. Compounds were easily detectable at 254 nm or 365 nm.

Flash column chromatography using silica gel 60 (0.040–0.063 mm, Merck, Darmstadt, Germany) was used in the purification of the synthesized compounds. Melting points (m.p.) were measured in a Köfler microscope (Wagner and Munz, Munich, Germany) and are uncorrected. ^1^H- and ^13^C-nuclear magnetic resonance (NMR) spectra were recorded at the University of Aveiro, Department of Chemistry in CDCl_3_ or DMSO-d_6_ (Deutero GmbH, Ely, UK) at room temperature on a Bruker Avance 300 spectrometer (300.13 MHz for ^1^H and 75.47 MHz for ^13^C, Bruker Biosciences Corporation, Billerica, MA, USA). Chemical shifts are expressed in δ (ppm) values relative to tetramethylsilane (TMS) as an internal reference. Coupling constants are reported in hertz (Hz). ^13^C-NMR assignments were made by bidimensional heteronuclear single quantum coherence (HSQC) and heteronuclear multiple bond correlation (HMBC) NMR experiments (long-range C, H coupling constants were optimized to 7 Hz) or by comparison with the assignments of similar molecules. High-resolution mass spectroscopy (HRMS) spectra were measured on a Bruker FTMS APEX III mass spectrometer (Bruker Corporation, Billerica, MA, USA) and recorded as electrospray ionization (ESI) mode in Centro de Apoio Cientifico e Tecnolóxico á Investigación (CACTI, University of Vigo, Pontevedra, Spain) or on an LTQ Orbitrap XL hybrid mass spectrometer (Thermo Fischer Scientific, Bremen, Germany) at CEMUP, University of Porto, Portugal. The following compounds were synthesized and purified by the described procedures.

#### 3.1.1. General Procedure for the Synthesis of Aminated Tetracyclic Thioxanthenes

To a suspension of 1-chloro-4-propoxy-9*H*-thioxanthen-9-one (**6**, 0.250 mg, 0.1 mmol) and the appropriate guanidine or urea derivative (**7**–**10**, 0.2 mmol) in methanol (25 mL), CuI (2 mg) and K_2_CO_3_ (0.1 mmol) were added and the suspension was heated at 100 °C for 48 h, in a sealed flask. The ^1^H and ^13^C NMR spectra and HRMS chromatograms for compounds **11**–**14** are present in the Supplementary Materials (Figures S1–S8).

##### Synthesis of 6-Propoxythiochromeno[4,3,2-de]quinazolin-2-amine (**11**)

Twenty-five percent as red needles. m.p. 180–182 ^o^C [chloroform (8):acetone (2)]. IR (KBr) νmax: 3462, 3297, 3157, 2963, 1638, 1608, 1569, 1558, 1545, 1502, 1453, 1436, 1405, 1391, 1368, 1318, 1261, 1232, 1152, 1123, 1100, 1064, 980, 906, 823, 800, 765, 726, 643, 607, 572, 539, 475. ^1^H NMR (CDCl_3_, 500.13 MHz) δ (ppm): 8.79 (1H, dd, J = 8.10 and 1.58 Hz, H8), 7.46 (1H, ddd, J = 8.05, 6.75 and 1.35 Hz, H6), 7.43 (1H, dd, J = 8.00 and 1.58 Hz, H5), 7.39 (1H, d, J = 9.00 Hz, H3), 7.36 (1H, ddd, J = 8.15, 6.70 and 1.53 Hz, H7), 7.32 (1H, d, J = 9.00 Hz, H2), 5.05 (2H, s, NH2), 4.12 (2H, t, J = 6.45 Hz, Ha), 1.90 (2H, st, J = 7.35 Hz, Hb), 1.12 (3H, t, J = 7.43 Hz, Hc). ^13^C NMR (CDCl_3_, 75.48 MHz) δ (ppm): 159.8 (C9), 159.6 (C1′), 149.2 (C1), 146.6 (C4), 136.4 (C10a), 131.6 (C6), 127.9 (C8), 127.7 (C8a), 126.7 (C4a), 126.4 (C7), 120.2 (C3), 120.1 (C2), 119.6 (C4a), 115.4 (C9a), 71.7 (Ca), 23.0 (Cb), 10.8 (Cc) (Figure S1). HRMS (ESI^+^): m/z [C_17_H_15_N_3_OS + H]^+^ calcd. for [C_17_H_16_N_3_OS]: 310.1009; found 310.0910 (Figure S2).

##### Synthesis of 6-Propoxythiochromeno[4,3,2-de]quinazoline (**12**)

Eleven percent as yellow needles. m.p. 89–91 ^o^C [chloroform (9):acetone (1)]. IR (KBr) νmax: 3443, 2922, 2852, 2360, 2342, 1570, 1560, 1530, 1502, 1442, 1369, 1344, 1281, 1262, 1210, 1101, 1069, 1028, 988, 831, 803, 750, 724, 669. ^1^H NMR (CDCl_3_, 300.13 MHz) δ (ppm): 9.02 (1H, s, H1′), 8.85 (1H, dd, J = 7.98 and 1.04 Hz, H8), 7.74 (1H, d, J = 9.06 Hz, H2), 7.53 (1H, d, J = 9.09 Hz, H3), 7.48 (1H, ddd, J = 8.04, 6.69 and 1.39 Hz, H6), 7.43 (1H, m, H5), 7.38 (1H, ddd, J = 8.07, 6.69 and 1.43 Hz, H7), 4.19 (2H, t, J = 6.41 Hz, Ha), 1.93 (2H, st, J = 7.26 Hz, Hb), 1.14 (3H, t, J = 7.41 Hz, Hc). ^13^C NMR (CDCl_3_, 75.48 MHz) δ (ppm): 158.2 (C9), 154.4 (C1′), 149.3 (C4), 146.9 (C1), 136.0 (C1a), 131.9 (C6), 128.0 (C8), 127.8 (C8a), 126.8 (C7), 126.7 (C5), 123.2 (C2), 120.6 (C9a), 119.0 (C3), 118.6 (C4a), 71.4 (Ca), 22.9 (Cb), 10.8 (Cc) (Figure S3). HRMS (ESI^+^): *m*/*z* [C_17_H_14_N_2_OS + H]^+^ calcd. for [C_17_H_15_N_2_OS]: 295.0900; found 295.0893 (Figure S4).

##### Synthesis of *N*-(2-Chlorobenzyl)-6-propoxythiochromeno[4,3,2-de]quinazolin-2-amine (**13**)

Forty-five percent as red needles. m.p. 166.9–168.0 °C (methanol). IR (KBr) νmax: 3245, 2962, 2936, 2880, 1611, 1553, 1533, 1473, 1439, 1401, 1351, 1335, 1316, 1260, 1251, 1229, 1075, 1049, 1039, 990, 819, 794, 761, 742, 721. ^1^H NMR (CDCl_3_, 300.13 MHz) δ (ppm): 8.77 (1H, dd, J = 7.89 and 1.16 Hz, H8), 7.38 (1H, d, J = 9.18 Hz, H3), 7.38 (7H, m, H5, H6, H7, H5′, H6′, H7′ and H8′), 7.20 (1H, d, J = 9.27 Hz, H2), 5.55 (1H, t, J = 5.49 Hz, NH), 4.90 (2H, d, J = 5.97 Hz, H2′), 4.11 (2H, t, J = 6.45 Hz, Ha), 1.89 (2H, st, J = 7.28 Hz, Hb), 1.12 (3H, t, J = 7.40 Hz, Hc). ^13^C NMR (CDCl_3_, 75.48 MHz) δ (ppm): 159.2 (C9), 159.0 (C1′), 149.2 (C1), 146.3 (C4), 137.32 (C4′), 136.3 (C10a), 133.7 (C3′), 131.4 (C6), 129.8 (C8′), 127.8 (C8), 127.0 (C8a), 119.5 (C4a), 71.8 (Ca), 43.5 (C3′), 23.0 (Cb), 10.8 (Cc), Car (C2, C3, C5, C7, C9a, C5′, C6′ and C7′) (Figure S5). HRMS (ESI^+^): m/z [C_24_H_20_ClN_3_OS + H]^+^ calcd. for [C_24_H_21_ClN_3_OS]: 434.1094; found 434.1099 (Figure S6).

##### Synthesis of (*E*)-6-Propoxy-*N*,3-di-*o*-tolylthiochromeno[4,3,2-de]quinazolin-2(3*H*)-imine (**14**)

Thirteen percent as red dust. m.p. 194.6–196.0 °C (chloroform:n-hexane). IR (KBr) νmax: 3423, 2964, 2935, 2876, 1601, 1578, 1542, 1467, 1421, 1384, 1337, 1317, 1263, 1124, 1080, 1070, 1043, 1023, 1004, 938, 806, 791, 774, 761, 749, 722, 671, 654, 584. ^1^H NMR (CDCl_3_, 300.13 MHz) δ (ppm): 8.44 (1H, dd, J = 8.19 and 0.98 Hz, H8), 7.51 (1H, m, H10′), 7.49 (1H, m, H6), 7.43 (3H, m, H4′, H11′ and H13′), 7.32 (2H, m, H3′ and H5′), 7.12 (3H, m, H5, H6′ and H12′), 6.99 (1H, d, J = 9.03 Hz, H3), 6.91 (1H, td, J = 6.48 and 2.05 Hz, H7), 6.13 (1H, d, J = 8.94 Hz, H2), 4.03 (2H, t, J = 6.39 Hz, Ha), 2.25 (3H, s, H15′), 2.08 (3H, s, H8′), 1.89 (2H, st, J = 7.24 Hz, Hb), 1.11 (3H, t, J = 7.40 Hz, Hc). ^13^C NMR (CDCl_3_, 75.48 MHz) δ (ppm): 157.5 (C9), 147.9 (C2′), 147.0 (C4), 140.0 (C1), 137.9 (C1′), 136.8 (C9′), 135.8 (C10a), 132.0 (C13′), 131.8 (C6′), 131.8 (C6), 131.2 (C8a), 129.8 (C6′), 129.2 (C4′), 129.1 (C8), 129.0 (C3′), 128.4 (C11′), 127.7 (C14′), 127.0 (C5′), 126.4 (C10′), 125.6 (C12′), 123.5 (C4a), 123.1 (C5), 121.9 (C7), 117.2 (C3), 111.7 (C9a), 109.6 (C2), 71.6 (Ca), 22.8 (Cb), 18.6 (C8′), 17.8 (C15′), 10.8 (Cc) (Figure S7). HRMS (ESI^+^): m/z [C_31_H_28_N_3_OS + H]^+^ calcd. for [C_31_H_29_N_3_OS]: 490.1953; found 490.1948 (Figure S8).

### 3.2. Biological Activity

To evaluate the biological activity of compounds **11**–**14**, three human tumor cell lines (European Collection of Cell Culture, Salisbury, Wiltshire, UK) were used: A375-C5 (melanoma), MCF-7 (breast adenocarcinoma) and NCI-H460 (non-small cell lung cancer). Cells were grown in RPMI-1640 culture medium (Biochrom, Berlin, Germany) supplemented with 5% heat-inactivated fetal bovine serum (FBS, Biochrom) and maintained in a humidified incubator at 37 °C with 5% CO_2_ (Hera Cell, Heraeus).

#### Antitumor Activity

The antitumor activity of compounds was accessed through the determination of the GI_50_ by the sulforhodamine B assay (SRB, Sigma-Aldrich). Tumor cells were seeded in 96-well plates (0.05 × 10^6^ cells/well) in complete culture medium and incubated at 37 °C for 24 h, allowing cell attachment. Cells were then incubated with two-fold serial dilutions of the test compounds, with concentrations ranging from 0 to 75 μM. Dimethyl sulfoxide-treated cells (DMSO), up to 0.25% concentration, were included as compounds solvent control. Forty-eight hours later, cells were fixed with 50% (*m*/*v*) trichloroacetic acid (Merck Millipore, Darmstadt, Germany), washed with distilled water and stained with SRB for 30 min at room temperature. The SRB-stained cells were washed 5 times with 1% (*v*/*v*) acetic acid (Merck Millipore) and afterwards dried at RT; SRB complexes were solubilized by adding 10 mM Tris-Base buffer (Sigma-Aldrich) for 30 min. Absorbance was measured at 515 nm in a microplate reader (BioTek Synergy 2, BioTek Instruments Inc., Winooski, VT, USA).The concentration that caused cell growth inhibition of 50% (GI_50_) was determined based on a dose–response curve obtained for each cell line with each test compound.

The non-tumor Human Pulmonary Alveolar Epithelial Cells (HPAEpiC) were used to assess the degree of selectivity of the compounds, as the ratio of GI_50_ in non-tumor cells over G_I50_ in cancer cells. A selectivity index value less than 2.0 indicates the general toxicity of the compound [32].

### 3.3. Photophysical Studies

The absorption spectra in dimethyl sulfoxide solutions were obtained on a Shimadzu UV-2501 PC spectrophotometer (1 cm path length quartz cell) and the emission spectra were recorded on a JASCO spectrofluorometer. Molar absorption coefficients were determined using 4 measurements in the concentration range 10^−3^–10^−6^ mol.L^−1^. Fluorescence quantum yields were determined by comparison with fluorescein in 0.1 M NaOH water solution as a fluorescence standard (φf = 90%) [29].

### 3.4. Fluorescence Microscopy and Flow Cytometry Analysis

To analyze compound-associated intracellular fluorescence, a total of 0.09 × 10^6^ cells were seeded on coverslips, allowing attachment for 24 h, and were treated with 5.66 μM of compound 11. After 48 h, cells were fixed with fresh 2% (*w/v*) paraformaldehyde (Sigma-Aldrich) in PBS for 12 min, followed by 3 times wash in PBS for 5 min and then permeabilized with 0.5% (*v/v*) Triton X-100 diluted in PBS for 7 min. DNA was stained with 2 µg/mL 4′,6-diamidino-2-phenylindole (DAPI, Sigma-Aldrich) diluted in Vectashield mounting medium (Vector, H-1000, Burlingame, CA, USA). Data analysis and image acquisition were performed using an Axio Observer Z.1 SD microscope (Carl Zeiss, Oberkochen, Germany), coupled to an AxioCam MR3, and with the Plan Apochromatic 63x/NA 1.4 objective. Fluorescence images were processed using ImageJ version 1.44 (http://rsb.info.nih.gov/ij/, accessed on 20 March 2021).

For flow cytometry analysis, cells exposed to compounds for 48 h were harvested, washed twice in PBS and the mean fluorescence intensity (MFI) of FITC-A was recorded in BD Accuri™ C6 Plus Flow cytometer (BD Biosciences, Qume Drive, San Jose, CA, USA). Cells treated with 1 µM of Rhodamine-123 were used as positive control. A total of 20.000 events per sample were collected. Data were analyzed with BD Accuri TM C6 Plus software, version 1.0.27.1 (www.bdbiosciences.com, accessed in 20 March 2021).

### 3.5. X-ray Crystallography

Diffraction data were collected at 291 K with a Gemini PX Ultra (Rigaku/Oxford, Neu-Isenburg, Germany) equipped with CuKα radiation (λ = 1.54184 Å). The structures were solved by direct methods using SHELXS-97 [33] and refined with SHELXL-97 [33]. Carbon, oxygen, nitrogen and sulfur atoms were refined anisotropically. Hydrogen atoms were either placed at their idealized positions using appropriate HFIX instructions in SHELXL, and included in subsequent refinement cycles, or were directly found from difference Fourier maps and were refined freely with isotropic displacement parameters. Full details of the data collection and refinement and tables of atomic coordinates, bond lengths and angles and torsion angles have been deposited with the Cambridge Crystallographic Data Centre (CCDC).

**11**. Crystals were monoclinic, space group P2_1_/c, cell volume was 2990.30(10) Å^3^ and unit cell dimensions were a = 12.1985(2) Å, b = 13.4082(3) Å and c = 18.8486(3) Å and β = 104.0758(18)°. There were two molecules in the asymmetric unit. The refinement converged to R (all data) = 7.26% and wR_2_ (all data) = 15.53%. CCDC 2065174.

**12**. Crystals were triclinic, space group P-1, cell volume was 966.33(18) Å^3^ and unit cell dimensions were a = 7.4849(5) Å, b = 11.7086(16) Å and c = 12.4900(13) Å and α = 63.635(12)°, β = 80.550(7)°, γ = 88.220(8)°. One molecule of B and one molecule of toluene (solvent) were found in the asymmetric unit. The refinement converged to R (all data) = 9.89% and wR_2_ (all data) = 13.55%. CCDC 2069933.

## 4. Conclusions

In this study, we report the synthesis of an unexpected tetracyclic thioxanthene scaffold, with very sparse descriptions in the literature. Different substitutions at the C-1′ position yielded four compounds, whose antitumor activity was studied for three different cell lines. Compounds **11** and **14** have been shown to be the best compounds in the three tested cell lines. The tetracyclic thioxanthenes were also studied for their fluorescence and it has been shown that, the higher the conjugation level, the more emissive and absorbent these compounds are in solution. In order to conjugate these two results, they were studied for their theranostic potential, and it was shown that compound **11** exhibits both high cytotoxic activity and a bright fluorescence property, making it a promising theranostic cancer drug candidate. Its easy intracellular uptake and accumulation at endosome-like structures could be used to target the respective subcellular structure, as a plausible therapeutic intervention to potentiate cancer cell death. Future work is needed to unveil the subcellular localization of the compound and its application to cancer therapy.

## Figures and Tables

**Table 1:** Amines used (**7**–**10**) and tetracyclic thioxanthenes (**11**–**14**) obtained and their isolated yields. All the reactions were carried out with 0.1 mmol of **6**, 0.2 mmol of the appropriate amine (**7**–**10**), 0.1 mmol of K_2_CO_3_ and 2 mg of CuI in methanol, at 100 °C, for 48 h.

Amine	Thioxanthene	Yield
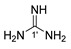 **7**	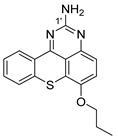 **11**	25%
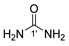 **8**	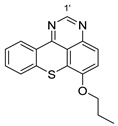 **12**	11%
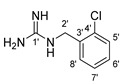 **9**	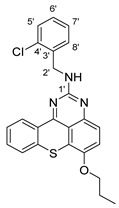 **13**	45%
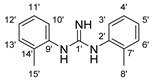 **10**	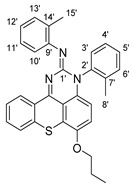 **14**	13%

**Table 2:** Cell growth inhibitory activity of compounds **11**–**14**.

Compound	Antitumor Activity (µM)		
	A375-C5	MCF-7	NCI-H460
**11**	6.48 ± 0.88	6.39 ± 0.49	5.66 ± 0.89
**12**	38.77 ± 5.93	33.82 ± 1.61	31.19 ± 1.53
**13**	22.88 ± 3.75	18.85 ± 1.42	16.97 ± 3.12
**14**	8.02 ± 3.59	9.04 ± 0.89	10.64 ± 0.31
Doxorubicin	0.016 ± 0.005	0.018 ± 0.003	0.024 ± 0.006

The antitumor activity is presented as the concentrations that cause 50% cell growth inhibition (GI_50_) after a continuous exposure for 48 h and represents mean ± SD from at least three independent experiments. Doxorubicin was used as positive control for the SRB assay.

**Table 3:** Selectivity index of compounds **11**–**14**.

Compound	HPAEpiC	Selectivity Index		
	(GI_50_, µM)	A375-C5	MCF-7	NCI-H460
**11**	26.75 ± 1.20	4.13	4.19	4.73
**12**	39.28 ± 0.25	1.01	1.16	1.26
**13**	127.90 ± 1.56	5.59	6.79	7.54
**14**	>150	-	-	-
Doxorubicin	0.054 ± 0.005	3.38	3.00	2.25

Results are expressed as mean ± SD of three independent experiments.

**Table 4:** Absorption and emission data in DMSO.

Compound	λ_max_ (nm)	log ε	λ_em_ (nm)	Stokes’ Shift (nm)	*Φ*_F_ % ^1^
**11**	456	3.67	548	92	29.0
**12**	394	3.72	483	89	50.7
**13**	457	3.72	548	91	31.3
**14**	480	4.00	560	80	<0.1

^1^ Determined by comparison with fluorescein (φf = 90% in water with NaOH 0.1 mol.L^−1^) [29].

## Data Availability

Not applicable.

## Supplementary Materials

The following are available online. Figure S1. ^1^H NMR (500.13 MHz, CDCl_3_) and ^13^C NMR (75.48 MHz, CDCl_3_) for compound **11**. Figure S2. Electrospray ESI data for compound **11**. Figure S3. ^1^H NMR (300.13 MHz, CDCl_3_) and ^13^C NMR (75.48 MHz, CDCl_3_) for compound **12**. Figure S4. Electrospray ESI data for compound **12**. Figure S5. ^1^H NMR (300.13 MHz, CDCl_3_) and ^13^C NMR (75.48 MHz, CDCl_3_) for compound **13**. Figure S6. Electrospray ESI data for compound **13**. Figure S7. ^1^H NMR (300.13 MHz, CDCl_3_) and ^13^C NMR (75.48 MHz, CDCl_3_) for compound **14**. Figure S8. Electrospray ESI data for compound **14**.
