# Supplementary material for: Tetracyclic Thioxanthene Derivatives: Studies on Fluorescence and Antitumor Activity
Source: Molecules. 2021 May 31;26(11):3315. doi: 10.3390/molecules26113315 (PMC8198043; doi:10.3390/molecules26113315)
Supplement: Supplementary file 1 [file molecules-26-03315-s001.zip › molecules-1183803-supplementary.pdf]

Supporting Information

Tetracyclic thioxanthene derivatives: studies on fluorescence and antitumor activity

Fernando Durães [1,2], Patrícia M. A. Silva [3], Pedro Novais [3,4], Isabel Amorim [5], Luís Gales [6,7], Cátia I. C. Esteves [8], Samuel Guieu [8, 9], Hassan Bousbaa [3,*], Madalena Pinto [1,2], and Emília Sousa [1, 2,*;]

1 Laboratory of Organic and Pharmaceutical Chemistry, Department of Chemical Sciences, Faculty of Pharmacy, University of Porto, Rua de Jorge Viterbo Ferreira, 228, 4050-313 Porto, Portugal; fduraes5@gmail.com;

2 CIIMAR-Interdisciplinary Centre of Marine and Environmental Research, University of Porto, Novo Edifício do Terminal de Cruzeiros do Porto de Leixões, Avenida General Norton de Matos, S/N, 4450-208 Matosinhos, Portugal; madalena@ff.up.pt

3 CESPU, Institute of Research and Advanced Training in Health Sciences and Technologies (IINFACTS), Rua Central de Gandra, 1317, 4585-116 Gandra, Portugal; hassan.bousbaa@iucs.cespu.pt; patricia.silva@cespu.pt; pedro.ha.novais@gmail.com

4 Department of Biochemistry, Faculty of Sciences, University of Porto, Rua do Campo Alegre, s/n, 4169-007 Porto, Portugal

5 GreenUPorto (Sustainable Agrifood Production) Research CenterDepartment of Biology, Faculty of Sciences, University of Porto, Rua do Campo Alegre, s/n, 4169-007 Porto, Portugal; mpamorim@fc.up.pt

6 Department of Molecular Biology, ICBAS - Instituto de Ciências Biomédicas Abel Salazar, University of Porto, Porto, Portugal; lgales@ibmc.up.pt

7 Bioengineering & Synthetic Microbiology, I3S – Instituto de Investigação e Inovação em Saúde, University of Porto, Porto, Portugal

8 LAQV-REQUIMTE, Department of Chemistry, University of Aveiro, 3810-193 Aveiro, Portugal; sguieu@ua.pt; catiaiesteves@ua.pt

9 CICECO-Aveiro Institute of Materials, Department of Chemistry, University of Aveiro, 3810-193 Aveiro, Portugal

* Correspondence: hassan.bousbaa@iucs.cespu.pt, esousa@ff.up.pt

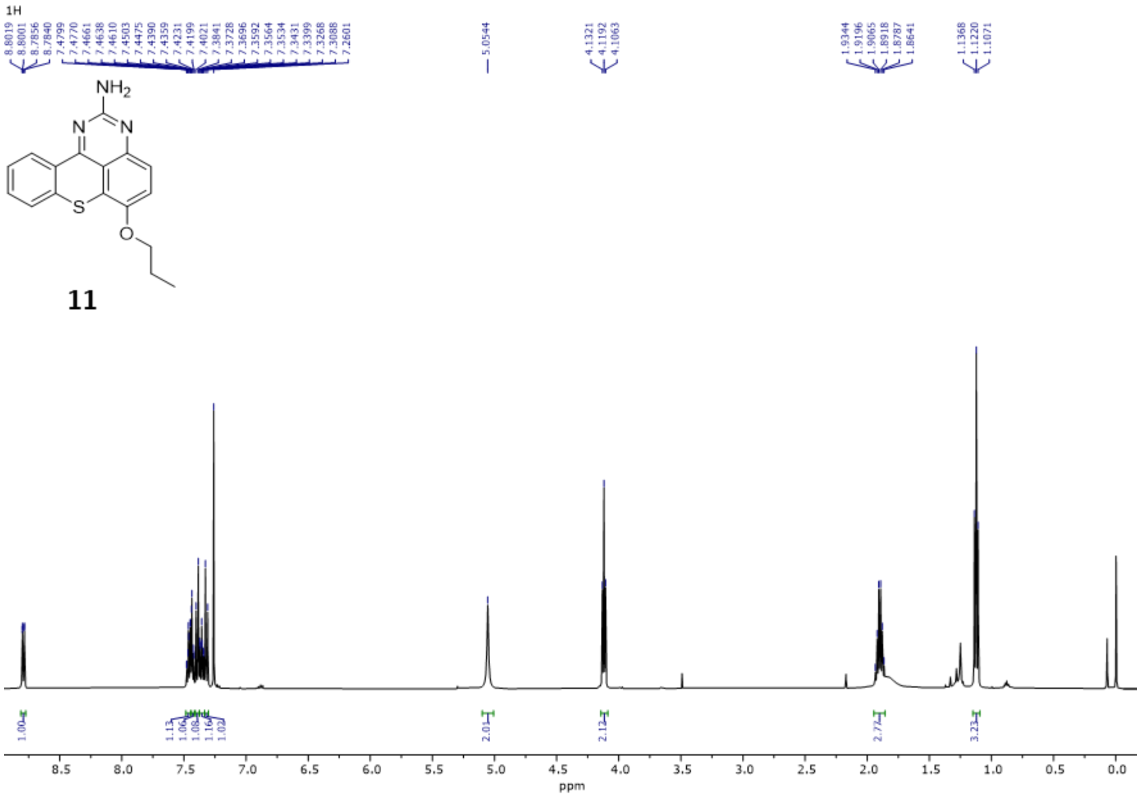

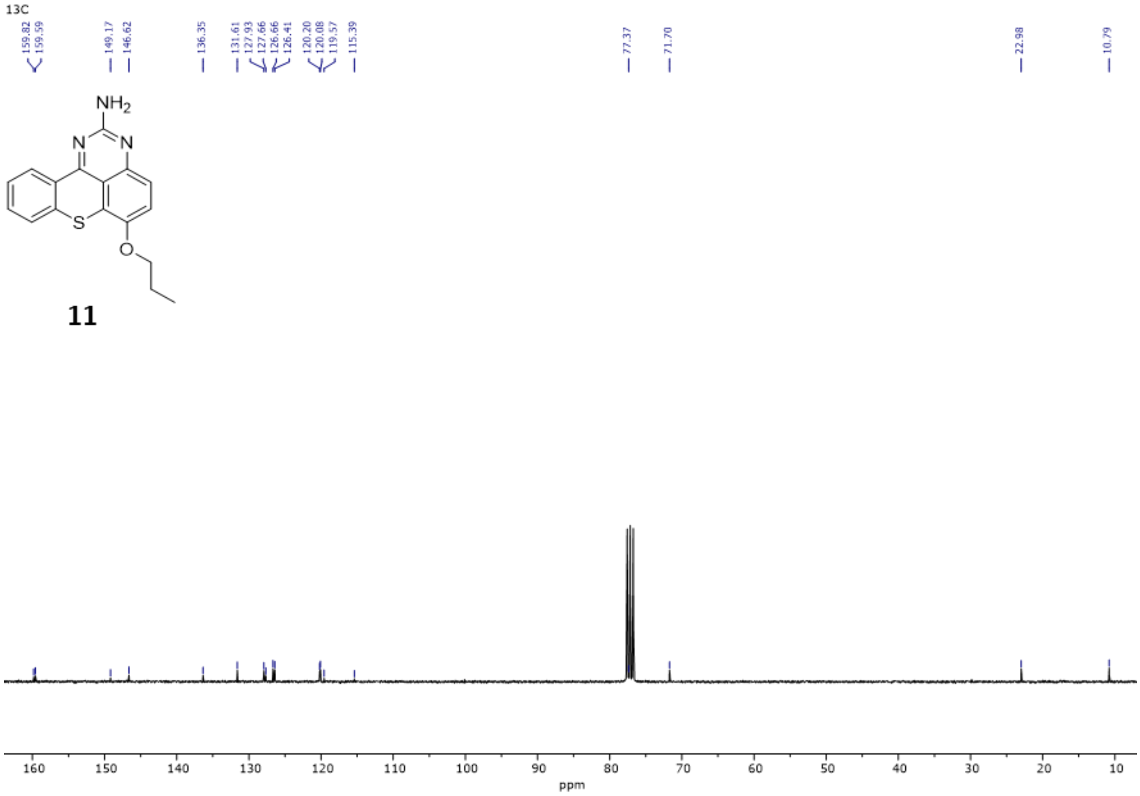

Figure S1. ^{1}H NMR (500.13 MHz, $CDCl_3$) and ^{13}C NMR (75.48 MHz, $CDCl_3$) for compound **11**.

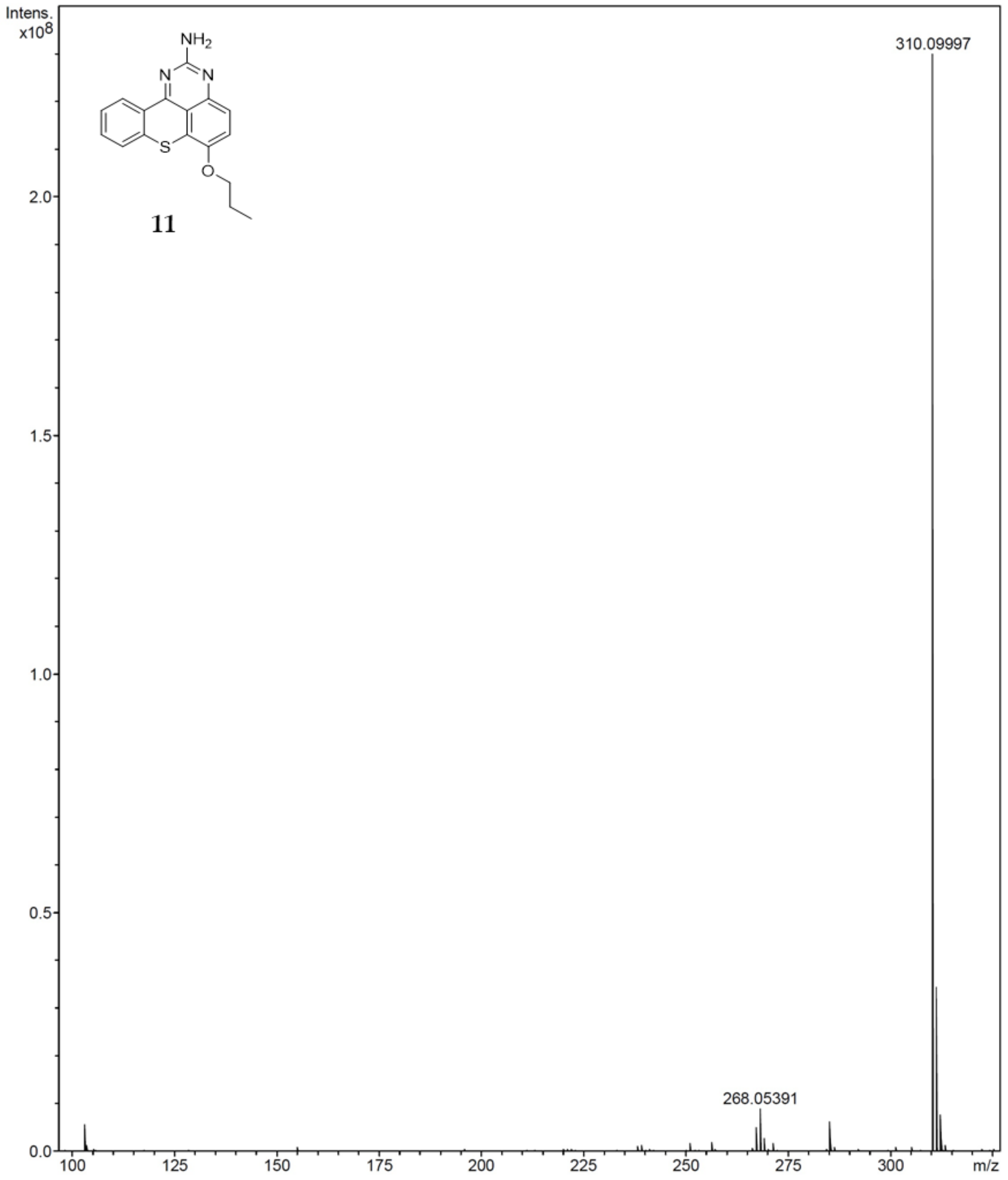

Meas. m/z	Formula	m/z	err [ppm]
310.09997	$C_{17}H_{16}N_3OS$	310.10065	2.87

Figure S2. Electrospray ESI data for compound **11**.

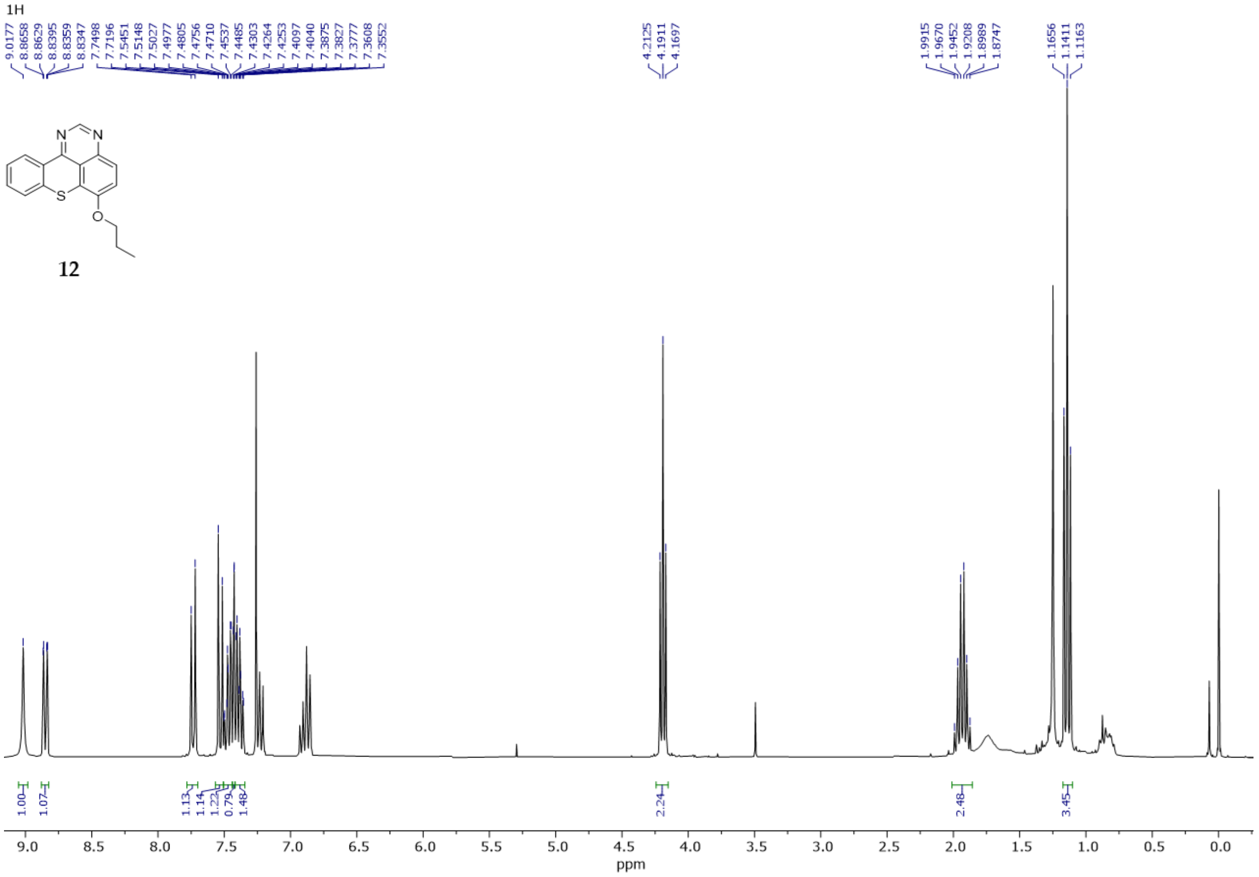

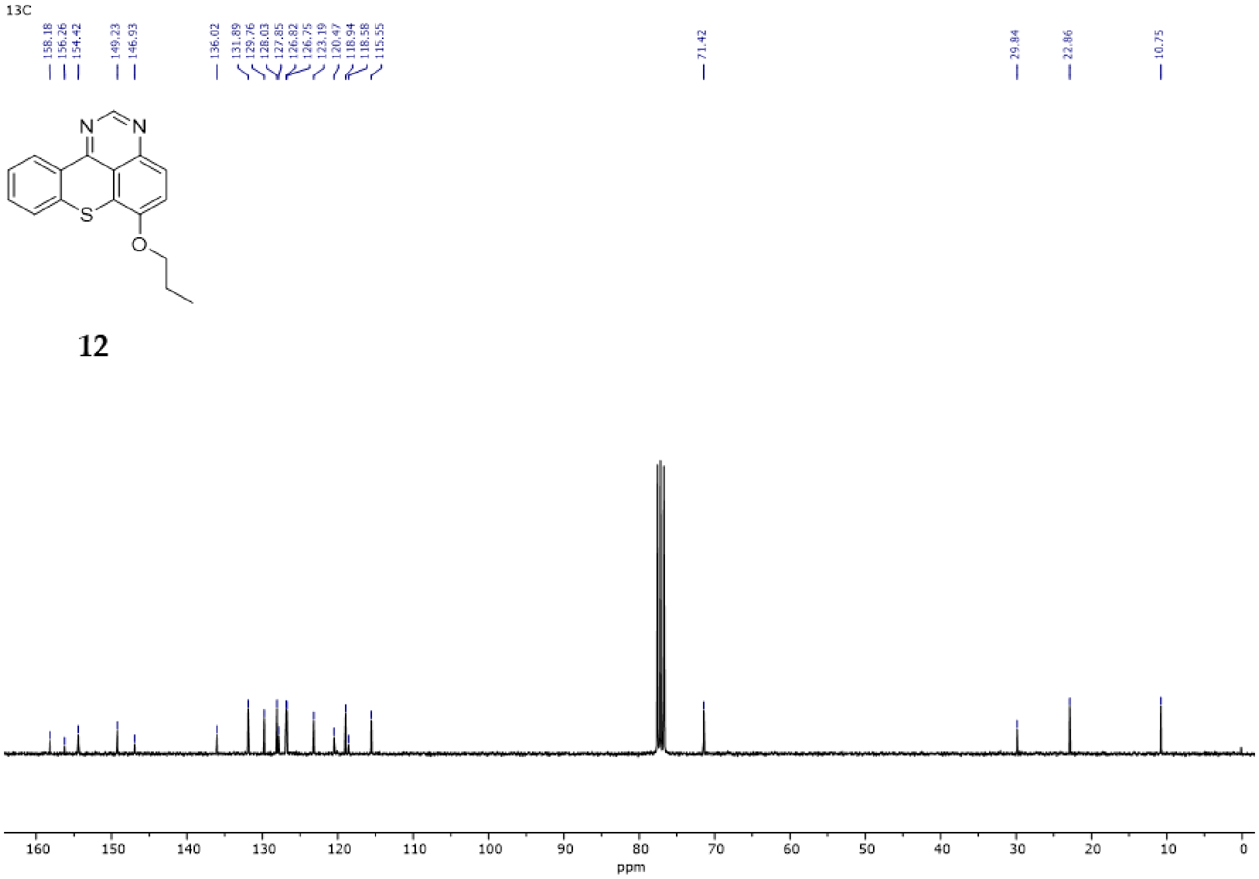

Figure S3. ^{1}H NMR (300.13 MHz, $CDCl_3$) and ^{13}C NMR (75.48 MHz, $CDCl_3$) for compound **12**.

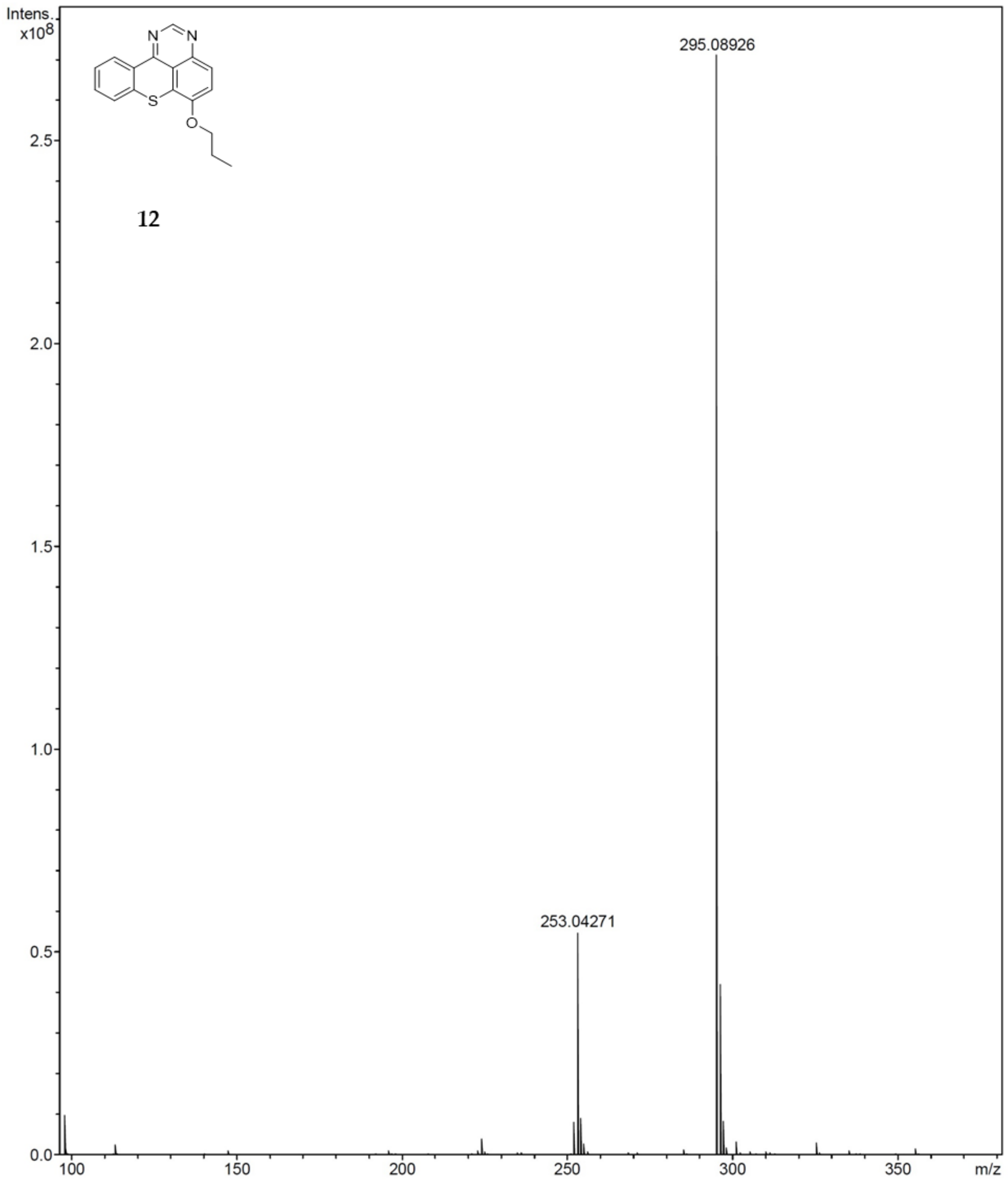

Meas. m/z	Formula	m/z	err [ppm]
295.08926	$C_{17}H_{15}N_2OS$	295.08995	2.36

Figure S4. Electrospray ESI data for compound **12**.

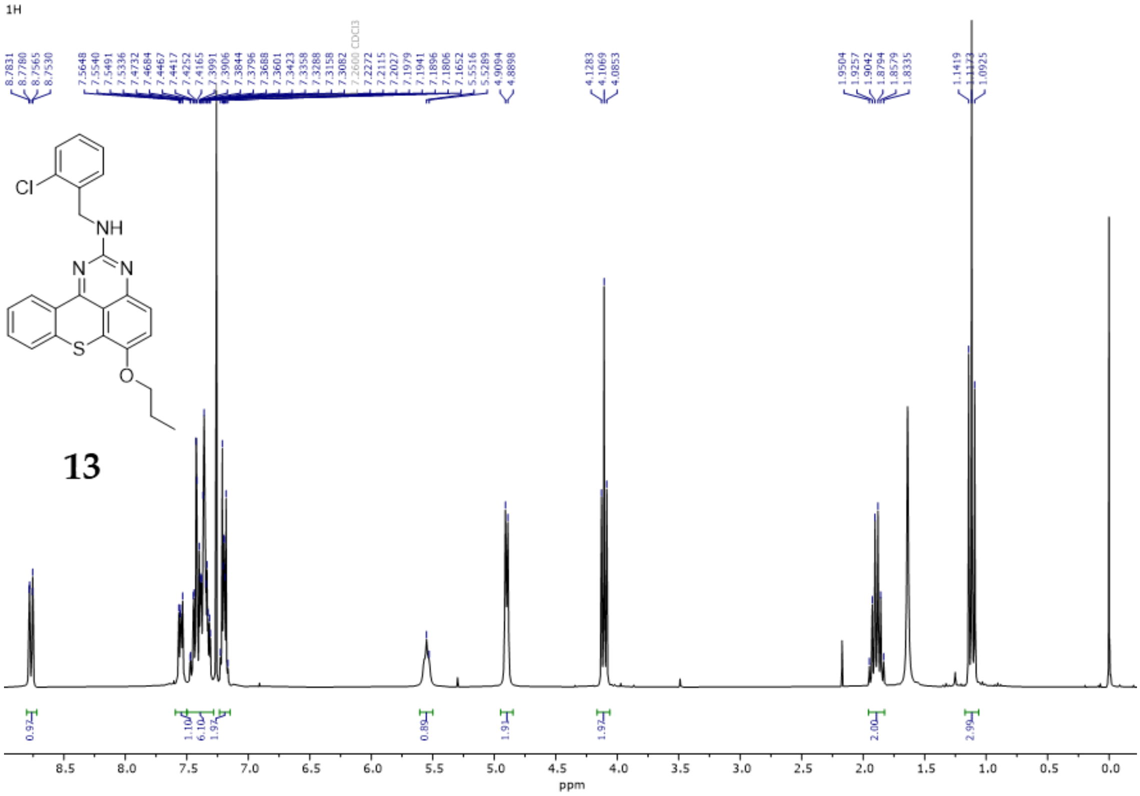

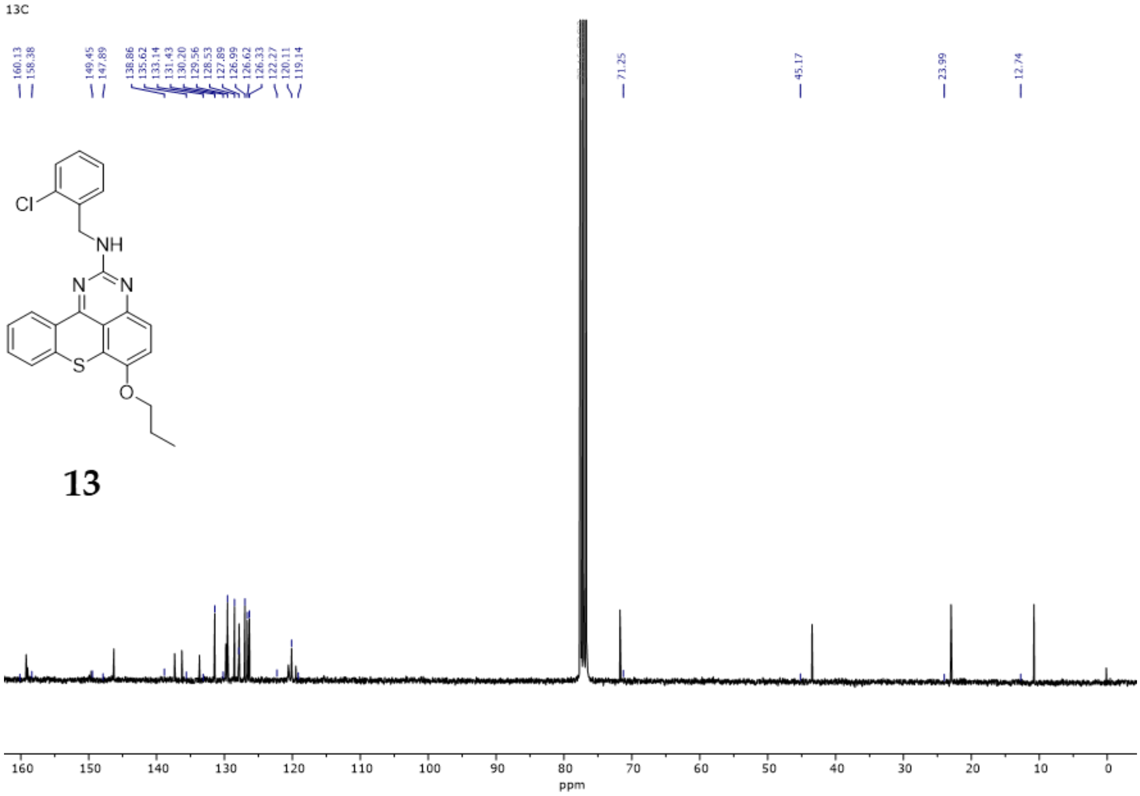

Figure S5. ^{1}H NMR (300.13 MHz, $CDCl_3$) and ^{13}C NMR (75.48 MHz, $CDCl_3$) for compound **13**.

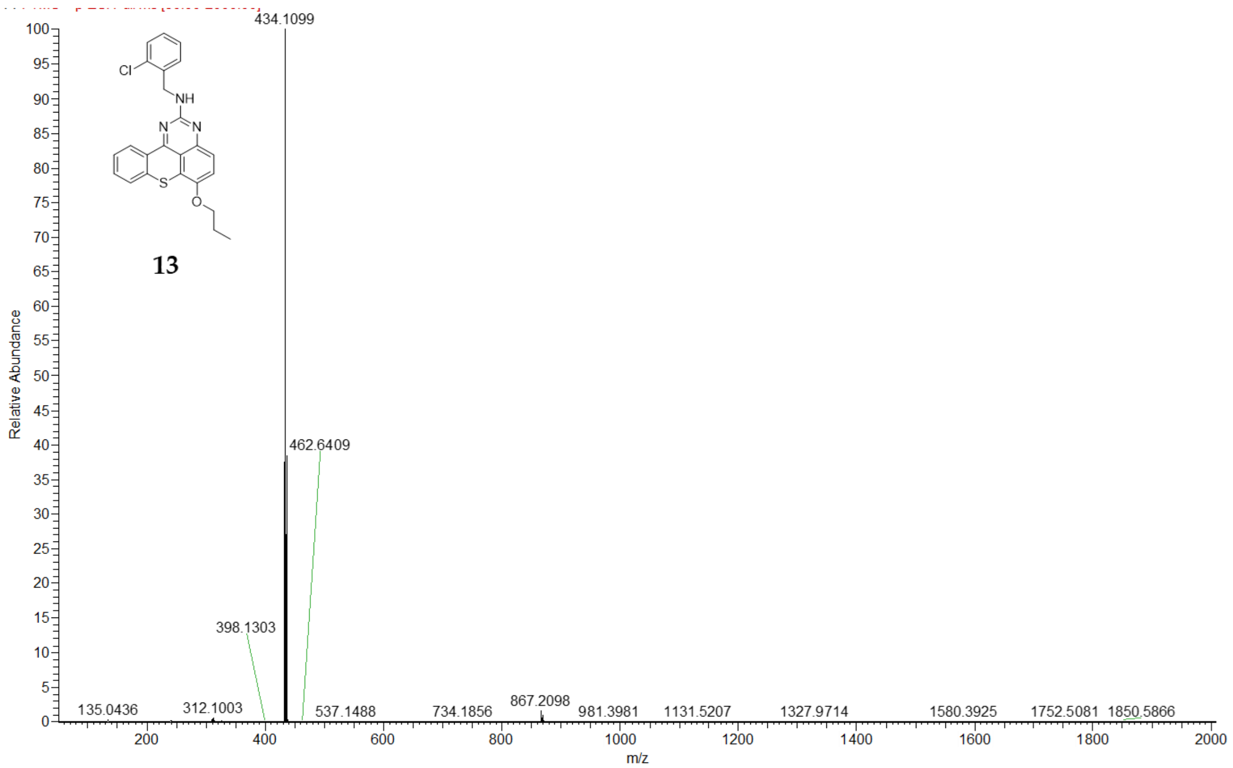

Meas. m/z	Formula	m/z	err [ppm]
434.1099	$C_{24}H_{20}ClN_3OS$	434.1094	1.1518

Figure S6. Electrospray ESI data for compound **13**.

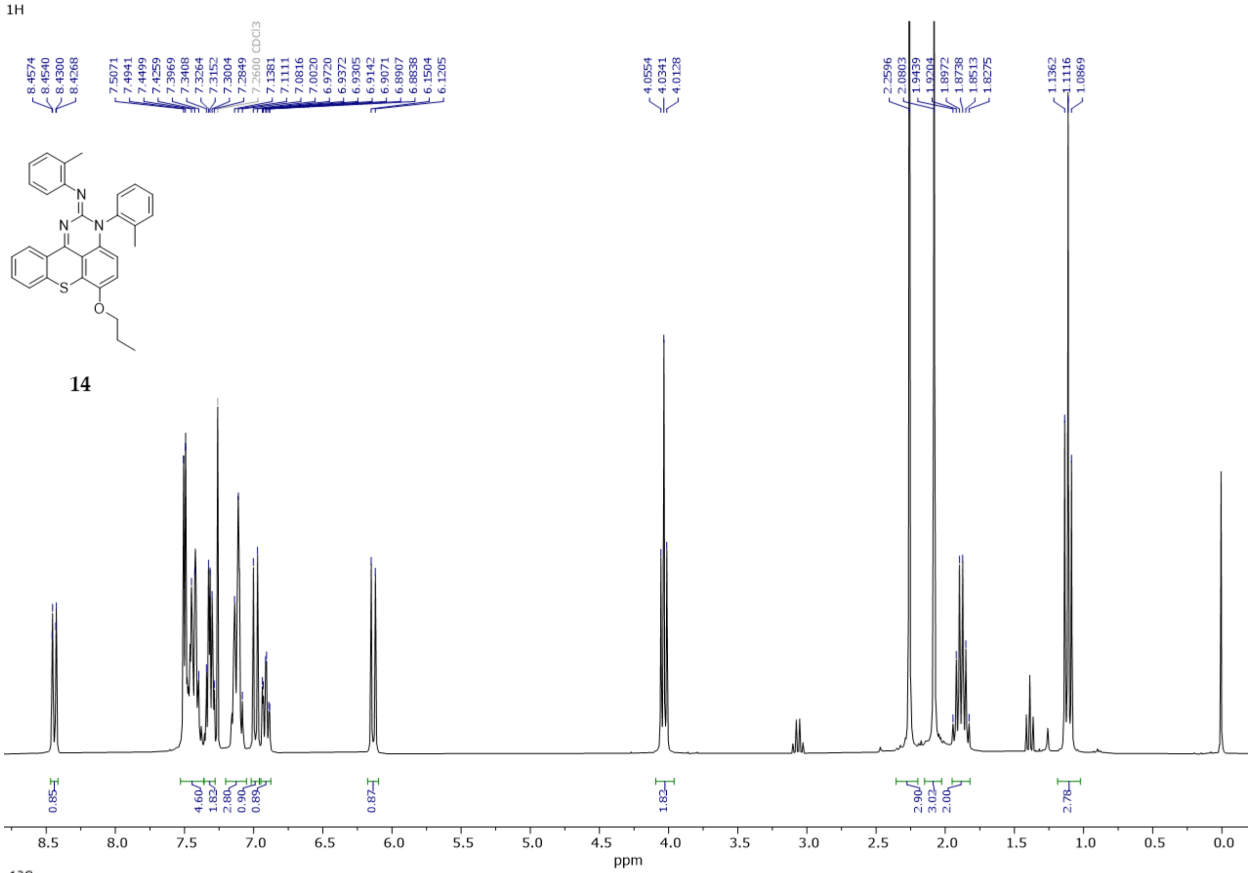

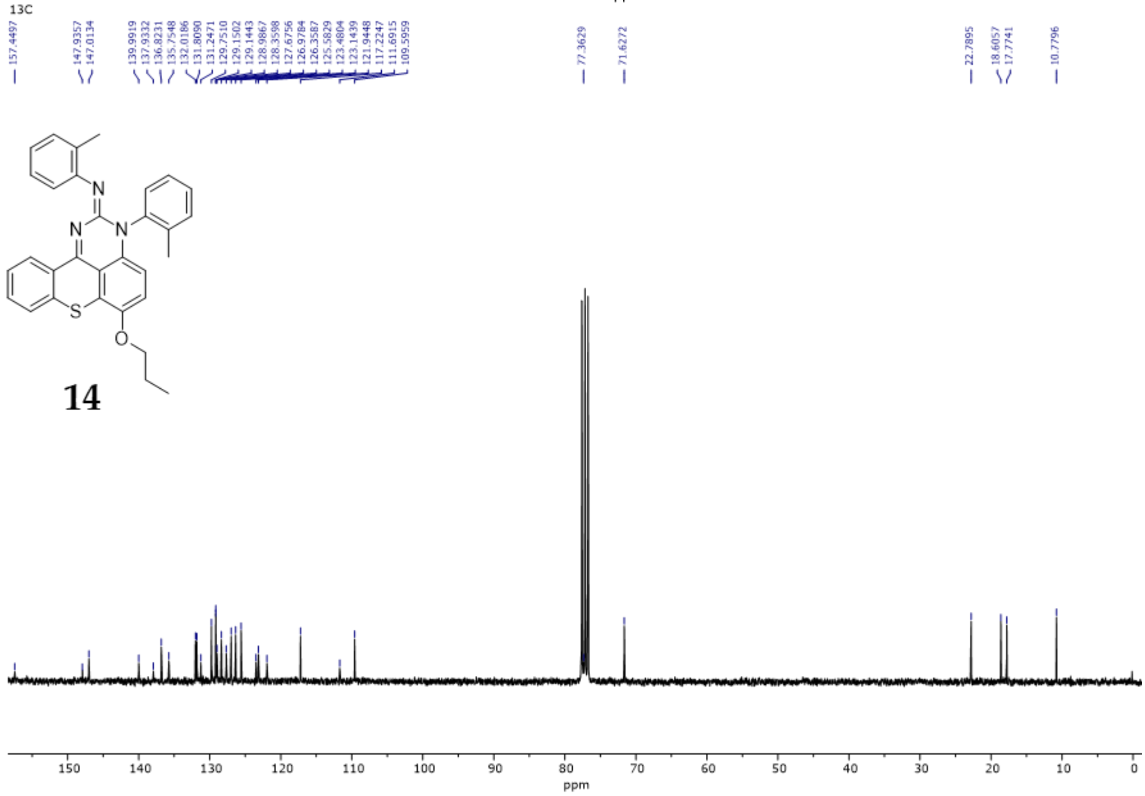

Figure S7. ^{1}H NMR (300.13 MHz, $CDCl_3$) and ^{13}C NMR (75.48 MHz, $CDCl_3$) for compound **14**.

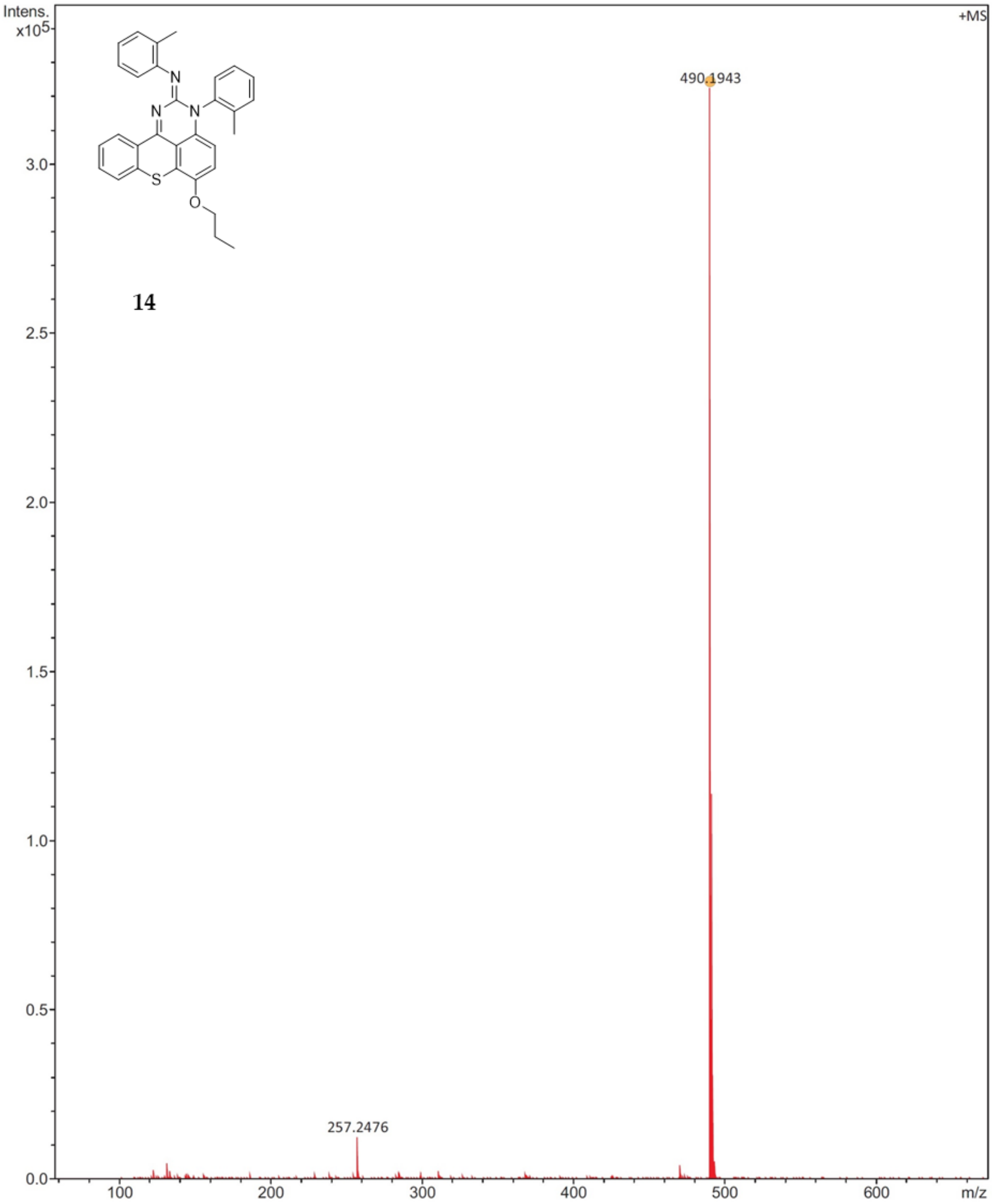

Meas. m/z	Formula	m/z	err [ppm]
490.1943	$C_{31}H_{28}N_3OS$	490.1948	1.0

Figure S8. Electrospray ESI data for compound **14**.
